# Hyaluronic Acid-Biocompatible Implant System for Rotator Cuff Augmentation

**DOI:** 10.1016/j.eats.2025.103990

**Published:** 2025-11-04

**Authors:** Raahil Patel, Andrew Moore, Collin Chase, Michael Kucharik, Viki Sochor, Humberto Cardona, Christopher Baker

**Affiliations:** a Department of Orthopaedic Surgery, University of South Florida Morsani College of Medicine, Tampa, Florida, U.S.A.; b Foundation for Orthopaedic Research and Education, Tampa, Florida, U.S.A.; c Florida Orthopaedic Institute, Florida, U.S.A.

## Abstract

Rotator cuff tears continue to have a high incidence of retear rates despite improvements in implants and repair techniques. The management of rotator cuff tears remains challenging because of the variability in both patient and tear pathology, without any universally accepted treatment to improve repair outcomes. Biologic patch augmentation has gained interest to enhance tendon healing, reduce retear rates, and restore the rotator cuff footprint. This Technical Note aims to describe a surgical technique using a hyaluronic acid-based biocompatible implant for both partial- and full-thickness rotator cuff tears. For partial-thickness tears, an onlay technique is used, placing the implant directly on the bursal surface of the tendon without formal suture repair. In full-thickness tears, the implant is applied over a completed standard rotator cuff repair construct to augment tendon-to-bone healing. This technique offers an alternative to currently utilized rotator cuff repair augmentation options.

Rotator cuff pathology is one of the most prevalent upper-extremity conditions managed by physicians. As arthroscopic techniques have improved, there has been a 1.6% increase in annual rotator cuff surgery in the United States for the past 2 decades.^1^ Rotator cuff retear after repair is a well-known risk with a rate that ranges from 11% to 94%, attributable to a variety of factors such as patient demographics and comorbidities, as well as tear size, retraction, and quality of tissue.^2^

Recently, there has been renewed interest in the use of biologic patch augmentations for rotator cuff repairs to improve outcomes and minimize failure rates. Studies have been published including various types of patch augmentations that can be divided into 3 main categories: xenografts, human grafts, and synthetic grafts.^3^ Although there remains limited data on the use of such grafts, there is promising evidence that patch augmentation may improve rotator cuff repair outcomes. A systematic review of randomized controlled trials of rotator cuff repair of large tears with patch augmentation by Orozco et al.^4^ found that patch augmentation may lead to lower retear rates, improved functional outcomes, and increased tendon thickness and footprint coverage. A meta-analysis by Warren et al.^5^ investigating the use of biocompatible patch augmentation in rotator cuff repairs showed a lower retear rate compared with traditional rotator cuff repair.

To address the limitations of traditional repair techniques, the use of a hyaluronic acid (HA)-based biocompatible implant has emerged as an adjunct to enhance tendon healing and repair durability. HA is a glycosaminoglycan present in the extracellular matrix that helps lubricate and cushion synovial joints. Less commonly known is that HA also influences cell migration, proliferation, and differentiation, making its biologic effects useful for therapeutic use.^6^^,^^7^ Typically used for intra-articular injections in orthopaedics, HA is also widely applied in wound care, ophthalmology, and aesthetic dermatology.^8^^,^^9^ The implant studied (Integrity; Anika Therapeutics) combines polyethylene terephthalate (20%) and Hyaff (80%), to form a solid HA derivative with a longer resorption time than liquid HA, which degrades rapidly. Hyaff has a proven safety record over decades and has been shown to significantly accelerate wound healing and reduce scar tissue formation.^10^, ^11^, ^12^ The advantages and disadvantages of this novel implant are described in Table 1.

**Table 1:** Advantages and Disadvantages of Hyaluronic Acid Biocompatible Implant System for Rotator Cuff Augmentation

Advantages	Disadvantages
Preclinical data shows robust thickening of collagen layer to augment rotator cuff	Novel design with limited long-term data
Hyaluronic acid implant can be used to protect tendon injuries without tissue loss	Not indicated for ligaments
Knit structure has high tensile strength	Cannot bridge a gap in tendon tears
Scaffold allows for cell infiltration and tissue remodeling	
Increased suture retention strength	

In this Technical Note, we describe a technique for patients with partial-thickness (PT) or full-thickness (FT) rotator cuff tears (RCTs) who are at greater risk for retear, requiring augmentation. We also provide a video (Video 1) that shows critical aspects of the surgical procedure for review. We hope to provide surgeons an option to reduce their failure rates of patients at risk for retear to improve patient outcomes while contributing to the advancement of rotator cuff repair techniques.

## Surgical Technique

All procedures are performed according to the senior author’s (R.P.) preference, with the patient in the lateral decubitus position using a hydraulic arm-positioning device (Spider Arm Holder; Smith & Nephew). An ultrasound-guided interscalene nerve block is administered preoperatively using bupivacaine to optimize intraoperative and early postoperative analgesia.

HA implant augmentation can be used in the management of both PT-RCTs and FT-RCTs, with distinct approaches depending on tear morphology. In cases of PT-RCTs, an “onlay” technique is used. The HA implant is applied directly to the bursal surface of the injured tendon without performing formal tendon repair. For FT-RCTs, the HA implant is applied after completion of the surgeon’s preferred techniques. Once an appropriate rotator cuff repair is secured, the implant is positioned on the bursal surface of the repair, spanning from the lateral aspect of the greater tuberosity to a point medial to the sutures of the medial row.

**Fig 1:**
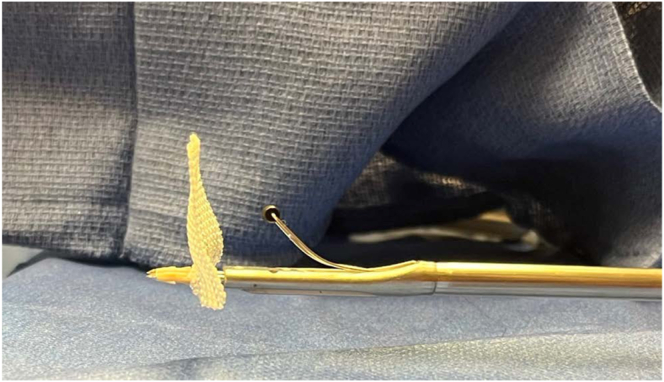
Modification of pushing wheel. The hyaluronic acid implant is preloaded onto the deployment device, with the implant and PEEK staple shown on the left and the deployment handle on the right. The author advises bending the pushing wheel (Anika Therapeutics) to 90° as shown, which facilitates both insertion and deployment of the implant.

**Fig 2:**
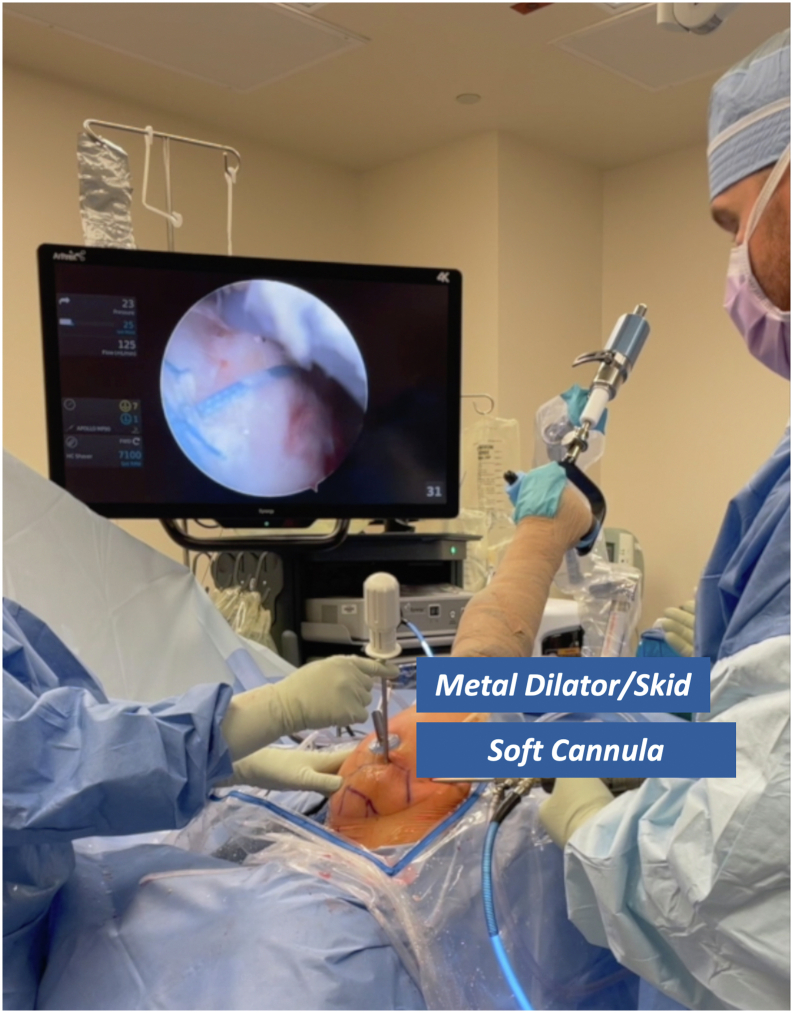
Soft cannula and metal dilator placement for hyaluronic acid implant. A 12-mm soft cannula (Arthrex) is introduced through the lateral working portal, followed by a metal dilator to facilitate implant passage. Image orientation: cranial left, caudal right, lateral superior, medial inferior. The patient is in the lateral decubitus position with the right arm secured in a hydraulic positioning device. The arthroscopic screen shows a completed double-row rotator cuff repair. The senior author recommends using the metal skid with a nonrigid cannula rather than a hard cannula/obturator to facilitate easier deployment.

**Fig 3:**
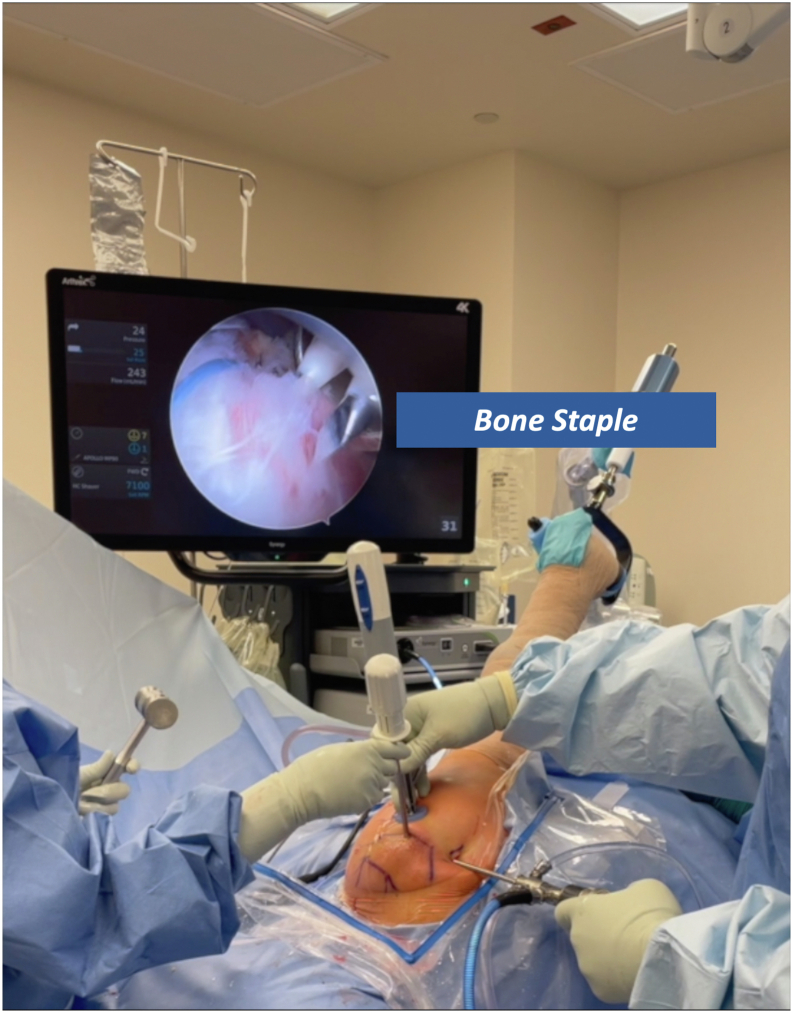
Use of the bone staple for HA implant insertion. The HA biocompatible implant is inserted with the PEEK bone staple (Anika Therapeutics) and impacted along the lateral row. Image orientation: cranial left, caudal right, lateral superior, medial inferior. The patient is in the lateral decubitus position with the right arm secured in a hydraulic positioning device. The arthroscopic view shows staple insertion along the lateral row of the repair. (HA, hyaluronic acid.)

**Fig 4:**
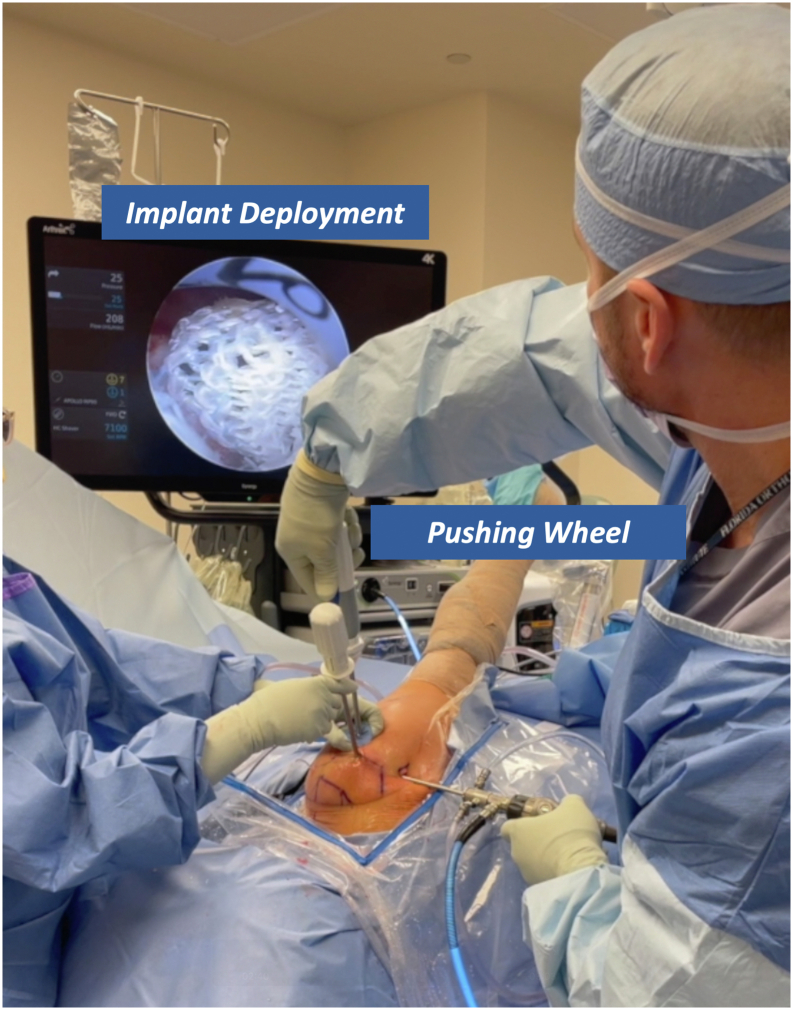
Deployment of the hyaluronic acid−biocompatible implant. Once the implant is fully seated, the metal dilator (Anika Therapeutics) is removed. The senior author recommends retracting the soft cannula while advancing the wheel, often repetitively, to deploy the implant over the rotator cuff repair. Image orientation: cranial left, caudal right, lateral superior, medial inferior. The patient is positioned in lateral decubitus with the right arm secured in a hydraulic positioning device. The arthroscopic view shows the implant draping over the double-row repair.

Before use, the deployment wheel (Anika Therapeutics) is bent, as shown in Figure 1, to assist with insertion and deployment of the implant. The PEEK (polyether ether ketone) staple (Anika Therapeutics) is then placed on the inserter handle and through the HA-biocompatible implant on the back table. Irrespective of the tear pattern, a 12-mm soft cannula (Arthrex) is passed through the lateral working portal, followed by a metal dilator to allow easy entry for the implant (Fig 2). The HA-biocompatible implant is inserted with the PEEK bone staple (Anika Therapeutics) and impacted along the lateral row (Fig 3). Once the implant is fully seated, the metal dilator (Anika Therapeutics) is removed. The senior author recommends pulling back on the soft cannula while pushing the wheel, sometimes repetitively, to deploy the implant over the rotator cuff repair (Fig 4). Technical pearls and pitfalls are described in Table 2.

**Table 2:** Pearls and Pitfalls of Hyaluronic Acid Biocompatible Implant System for Rotator Cuff Augmentation

Pearls	Pitfalls
Deployment skid can be used with a non-rigid cannula instead of hard cannula/obturator	Inadequate exposure or room in subacromial space may increase difficulty placing implant
Modify pushing wheel to allow for easier release	Inappropriate seating may hinder footprint coverage
Lateral first fixation with anchors eases placement via a single portal, while medial first fixation is preferred when using the 2-portal technique	Lack of modification of pushing wheel may hinder deployment
Soft cannula may provide better visualization	

**Fig 5:**
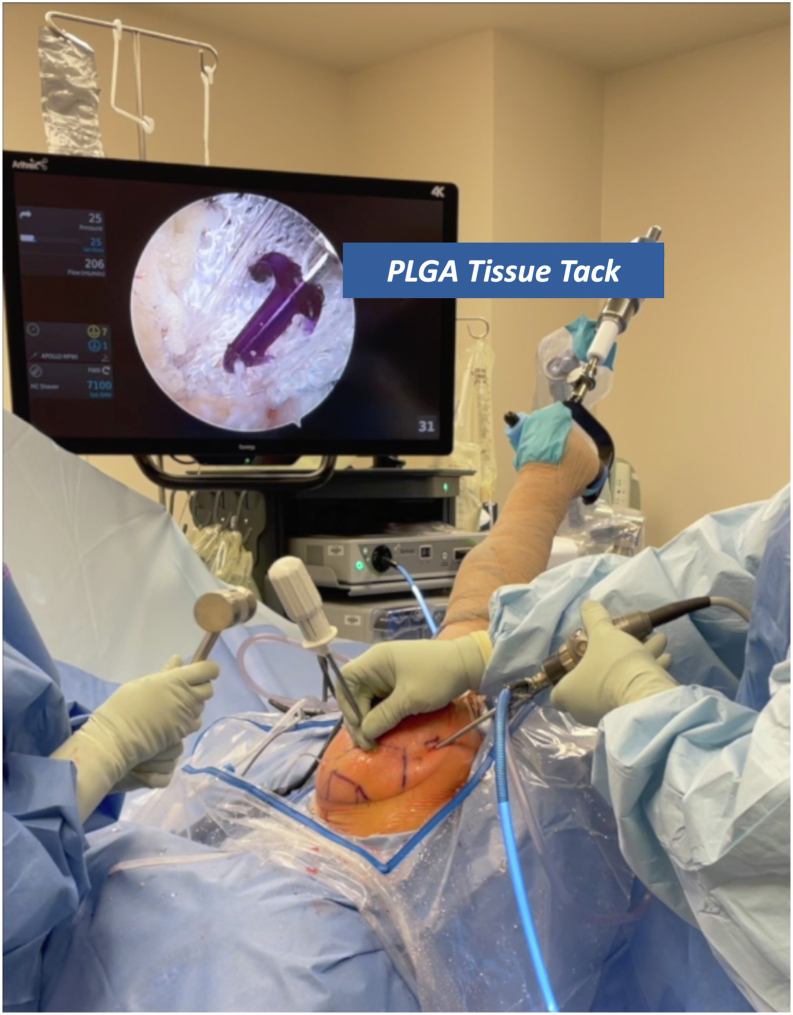
Insertion of the poly(lactic-co-glycolic) acid tissue tack. In the 2-portal technique, the tack insertion device is introduced through the lateral acromial portal, the same portal used for medial row anchor placement. The implant is fixated medially to the suture row to fully cover the rotator cuff repair. By retracting the deployment wheel, the PEEK inserter handle is removed, and remaining poly(lactic-co-glycolic) acid tacks are placed from medial to lateral and anterior to posterior until the implant is fully seated. Image orientation: cranial left, caudal right, lateral superior, medial inferior. The patient is in the lateral decubitus position with the right arm secured in a hydraulic positioning device. The arthroscopic view shows the anchor being placed along the implant border.

The implant is then secured using soft-tissue fixation tacks (Anika Therapeutics) composed of poly(lactic-co-glycolic) acid, ensuring secure fixation to the underlying tendon. This method preserves native tendon integrity and provides a biologic scaffold intended to promote healing. The tack insertion device is placed thru the lateral acromial portal used to insert the medial row suture anchors. The implant can then be fixated medially to the medial suture row to cover the entirety of the rotator cuff repair (Fig 5). By pulling back on the deployment wheel, the PEEK inserter handle can be removed and any remaining poly(lactic-co-glycolic) acid tacks can be secured medial to lateral and anterior to posterior. This can be continued until the implant is fully seated.

Postoperative rehabilitation protocols are tailored to the type of repair performed. For PT-RCT “onlay” repairs, patients discontinue sling use on postoperative day 3 and initiate hand, wrist, and elbow range-of-motion exercises, along with passive shoulder motion such as pendulums and table slides. Abduction and overhead elevation are restricted until 6 weeks postoperatively. Isometric strengthening for internal and external rotation with the arm at the side begins at 2 weeks. Full passive range of motion begins at 6 weeks with progressive resistance in all planes introduced at 3 months as patients work toward full active shoulder motion. For FT-RCT repairs with augmentation, the postoperative management is dictated per the surgeon’s preferred rotator cuff protocol based on the injury pattern.

## Discussion

The use of HA-based implant augmentation in rotator cuff repair has emerged as a promising biologic strategy to enhance tendon healing in both PT and FT tears. This technique guide outlines 2 distinct applications—onlay augmentation for PT tears and bursal-sided augmentation after standard repair for PT or FT tears. The senior author’s preference is to use the implant for young patients with atraumatic rotator cuff tears, individuals with chronic retracted tears, and cases in which there is significant degenerative tissue present.

From a biomechanical standpoint, augmentation supports improved footprint coverage—a critical determinant of tendon-to-bone healing success. The structure of the implant offers high tensile strength and suture retention as well as tear resistance. Especially in the context of limited native tendon quality or retracted tears, supplementing the repair construct with an implant or patch may help restore continuity across the tuberosity and reduce insertional strain. These benefits are especially relevant during the vulnerable early postoperative period. From a biologic standpoint, HA promotes cell proliferation^13^ and has potential to create an anti-inflammatory environment. In a controlled laboratory study, local application of a HA implant directly to the overlying injured tendon site in a rabbit model showed an increased healing response, increased biomechanical strength at early time points, and enhanced chondroid formation and collagen maturity at the tendon-bone interface. The mechanism appears to involve activation of mesenchymal stem cells and upregulation of cartilage-related genes, suggesting a potential role for HA as a surgical adjunct to promote healing after rotator cuff repair.^14^ However, the human clinical data of HA implant applications remain limited. This report shows the clinical application of a HA structural implant for rotator cuff augmentation. The objective of this technique article was to increase familiarity with the implant and highlight the expected postoperative appearances. These techniques may help refine biologically supported rotator cuff repair, with the potential to optimize healing conditions and functional recovery in higher-risk patients. This approach may ultimately contribute to improved long-term outcomes and reduced failure rates in rotator cuff repair.

## Disclosures

All authors (R.P., A.M., C.C., M.K., V.S., H.C., C.B.) declare that they have no known competing financial interests or personal relationships that could have appeared to influence the work reported in this paper.

## Supplementary Data

Video 1 This video shows the step-by-step technique for deploying a hyaluronic acid (HA)-biocompatible implant as an adjunct to rotator cuff repair. Using a 2-portal approach, a lateral working portal and a posterolateral viewing portal are established in the right arm to allow precise implant passage and fixation. The deployment sequence is shown in detail, including cannula placement, implant advancement, and fixation with PEEK staples and poly(lactic-co-glycolic) acid tacks to ensure secure coverage of the repaired tendon. A representative patient case is presented, highlighting the intraoperative technique and immediate postoperative construct. Follow-up imaging is provided, with ultrasound at 3 months revealing early incorporation of the implant and magnetic resonance imaging at 6 months confirming progressive tendon healing and integration of the scaffold. The video highlights key technical pearls to optimize implant handling, fixation, and biologic augmentation for rotator cuff repairs.
